# 

**Published:** 1926

**Authors:** 


### The Medical Library of the University of Bristol,

WITH WHICH IS INCORPORATED

**The Library of the Bristol Medico-Chirurgical Society.**

---

*The following donations have been received since the publication of the List in March,* 1926.

June, 1926.

| | | |
|---|---|---|
| Bureau for Increasing Use of Quinine (1) | .. | 1 volume. |
| Carnegie Institution of Washington (2) | .. | 1 ,, |
| Dr. Carey F. Coombs (3) | .. .. .. | 1 ,, |
| A. T. McClintock Memorial Foundation (4) | .. | 1 ,, |
| Michell Clarke Memorial Fund (5) | .. .. | 1 ,, |
| Professor J. A. Nixon (6) | .. .. .. | 2 volumes |
| Dr. W. K. Sibley (7) | .. .. .. .. | 1 volume |
| Smithsonian Institution (8) | .. .. .. | 1 ,, |
| Mr. W. S. V. Stock (9) | .. .. .. .. | 1 ,, |
| Dr. J. O. Symes (10) | .. .. .. .. | 1 ,, |

Unbound periodicals have been received from Professor Hey Groves, Dr. G. Parker and Dr. R. H. Burnett.

### THE ONE HUNDRED AND SIXTEENTH LIST OF BOOKS.

The figures in round brackets refer to the figures after the names of the donors. The books to which no such figures are attached have either been bought from the Library Fund or received through the *Journal.*

| | | | |
|---|---|---|---|
| **Belfrage, S. H.** | *What's Best to Eat?* | | 1926 |
| **Bousfield, P.** | *The Pathology, Diagnosis and Treatment of Functional Nervous Diseases* | | 1926 |
| **Brunschwig, H.** | *The Book of Cirurgia* | | 1923 |
| **Burrows, H.** | *The Muscular System* | 2nd Ed. | 1926 |
| **Cameron, H. C.** | *Diseases of Children* | | 1926 |
| **Campbell, W. R. and MacLeod, J. J. R.** | *Insulin.* | | 1925 |
| **Carpenter, T. M.** | *Human Metabolism with Enemata of Alcohol Dextrose and Levulose* | (2) | 1925 |
| *Chininum Scriptiones Collectœ Anno* 1924 | | (1) | 1925 |
| **Collins, E.T., and Mayou, M. S.** | *Pathology and Bacteriology of the Eye* | 2nd Ed. | 1925 |
| **Coombs, C. F.** | *Ætiology of Cardiac Disease* (*Long Fox Memorial Lecture*) | (3) | 1926 |
| **Colwell, H. A., and Wakeley, C. P. G.** | *Introduction to Study of X-Rays and Radium* | | 1926 |

| | | |
|---|---|---|
| **Cushing, H.** .. .. .. | *Studies in Intracranial Physiology and Surgery* | 1926 |
| **Dundas-Grant, Sir J.** .. | *Diagnosis and Treatment of Tuberculosis and Cancer of the Larynx* .. .. .. .. .. .. .. | 1926 |
| **Elsberg, C. A.** .. .. | *Tumors of the Spinal Chord* .. .. .. .. .. (5) | 1925 |
| **Evans, G. L.** .. .. | *Recent Advances in Physiology* .. .. .. .. | 1925 |
| **Fano, G.** .. .. .. | *Brain and Heart* .. .. .. .. .. .. .. .. .. | 1926 |
| **Fraser, J.** .. .. .. | *Surgery of Childhood.* 2 Vols. .. .. .. .. .. | 1926 |
| **Gordon, R. G.** .. .. | *Personality* .. .. .. .. .. .. .. .. .. .. | 1926 |
| **Gregory, H. H. C.** .. | *Infant Welfare* .. .. .. .. .. .. .. .. .. .. | 1926 |
| **Harman, N. B.** .. .. | *Aids to Ophthalmology* .. .. .. .. .. 6th Ed. | 1923 |
| **Haultain, W. F. T.** .. | *Handbook of Midwifery and Gynæcology* .. .. | 1926 |
| **Heatherley, F.** .. .. | *Modern Methods in Heart Disease* .. 2nd Ed. | 1926 |
| **Hernaman-Johnson, F.** | *Radiotherapy in Relation to General Medicine* .. | 1926 |
| *Hints to Patients on the Home Treatment of Functional Disorders of the Heart* | | — |
| **Howell, W. H.** .. .. | *Textbook of Physiology* .. .. .. .. .. 9th Ed. | 1925 |
| **Jack, W. R.** .. .. | *Wheeler's Handbook of Medicine* .. .. 7th Ed. | 1924 |
| **Leeuwen, W. S. V.** .. | *Allergic Diseases* .. .. .. .. .. .. .. .. (10) | 1925 |
| **McClintock, A. T.** .. | *Pleomorphism in Bacterial Protoplasm.* Vol. I. (4) | 1925 |
| **Mackenzie, Sir J.** .. | *Basis of Vital Activity* .. .. .. .. .. .. .. .. | 1926 |
| **MacLeod, J. J. R.** .. | *Physiology and Bio-chemistry in Modern Medicine* 4th Ed. | 1925 |
| **Medical Annual** .. .. | .. .. .. .. .. .. .. .. .. .. .. .. .. .. .. | 1926 |
| **Monrad-Krohn, G. H.** | *Clinical Examination of the Nervous System* 3rd Ed. | 1926 |
| **Morton, D.** .. .. .. | *Invalid Diet* .. .. .. .. .. .. .. .. .. .. .. | 1926 |
| **Muybridge, E.** .. .. | *The Human Figure in Motion* .. .. .. (9) | 1901 |
| **Perkins, G.** .. .. .. | *Diagnosis, Treatment and End Results of Tuberculous Disease of the Hip Joint* .. .. .. | 1926 |
| **de Poorter, A.** .. .. | *Catalogue des Manuscrits* .. .. .. .. .. .. | 1924 |
| **Price, F. W.** [Ed.] .. | *Textbook of the Practice of Medicine* .. 2nd Ed. | 1926 |
| **Roaf, H. E.** .. .. | *Textbook of Physiology* .. .. .. .. .. .. .. .. | 1924 |
| **Ryle, J. A.** .. .. .. | *Gastric Function in Health and Disease* .. .. | 1926 |
| **Sibley, W. K.** .. .. | *Treatment of the Diseases of the Skin* 3rd Ed. (7) | 1920 |
| **Sigerist, H. E.** [Ed.] | *Fasciculus Medicinæ of Johannes Ketham.* Vol. I., Vol. II., i., Vol. II., ii. .. .. .. .. .. .. | 1924-5 |
| **Singer, C.** .. .. .. | *Earliest Printed Syphilis Literature* .. .. .. | 1925 |
| **Singer, C.** .. .. .. | *Evolution of Anatomy* .. .. .. .. .. .. .. .. | 1925 |
| **Starling, E. H.** .. .. | *Principles of Human Physiology* .. .. 4th Ed. | 1926 |
| **Stewart, G. N.** .. .. | *Manual of Physiology* .. .. .. .. .. 8th Ed. | 1918 |
| **Ward, E.** .. .. .. | *Favourite Prescriptions* .. .. .. .. .. .. .. .. | 1926 |
| **Wilson, S. A. K.** .. | *Aphasia* .. .. .. .. .. .. .. .. .. .. .. .. .. | 1926 |

TRANSACTIONS, REPORTS, JOURNALS, ETC.

| | |
|---|---|
| Annual Report of the Smithsonian Institute .. .. .. .. .. .. (8) | 1924 |
| British Journal of Surgery, April, 1926 .. .. .. .. .. .. .. .. .. .. | 1926 |
| Cassel Hospital. Medical Report .. .. .. .. .. .. .. .. .. .. .. (6) | 1924 |
| Cassel Hospital. Medical Report .. .. .. .. .. .. .. .. .. .. .. (6) | 1925 |
| Studies from the Laboratories of the Philadelphia General Hospital. Vol. 3 | 1925 |
| Surgical Clinics of North America. Vol. 5, December .. .. .. .. .. | 1925 |
| ,, ,, ,, ,, ,, Vol. 6, February .. .. .. .. .. | 1926 |
| ,, ,, ,, ,, ,, Vol. 6, April .. .. .. .. .. .. | 1926 |

### Local Medical Notes.

UNIVERSITY NEWS.

The Postgraduate Committee of the University has arranged for a course of meetings to be held at the Bristol General Hospital every Wednesday at 3.0 p.m., and it is hoped that many practitioners will join.

EXAMINATION RESULTS.

**University of Bristol.**—Students of the University have recently passed the following examinations :—

M.B., Ch.B. (*Part I.*).—*Second Examination :* J. F. Coates, E. M. D. M. Collinson, J. L. W. Davies, J. J. J. Giraldi, R. D. Jones, R. P. Lucas, H. M. Strover, S. C. Wake. *In Elementary Anatomy* (*Completing Examination*) *:* J. L. Delicati. *In Elementary Anatomy only :* T. Evans, G. F. Langley, A. J. B. Miall, A. M. B. Walker. *In Organic Chemistry* (*Completing Examination*) *:* T. Evans, R. M. Hickman, G. F. Langley.

M.B., Ch.B. (*Part II.*).—*Second Examination :* I. J. Armstrong, R. A. S. Cory (with distinction in Physiology), A. A. Dowling, G. R. Fells, A. C. Fisher (with distinction in Anatomy), N. D. Gerrish, A. D. James, R. J. Krogh (with distinction in Anatomy and Physiology), N. L. Price, K. H. Pridie, H. D. Pyke, N. C. Tanner, L. E. Vine, G. J. West.

M.D.—E. Casson, M.B., Ch.B., by dissertation on "Primitive Instincts in Insanity."

Diploma in Dental Surgery.—*First Professional Examination : In Physiology and Anatomy* (*Completing Examination*) *:* H. J. Phillips. *In Dental Anatomy only :* H. G. Clapp, C. R. H. Edwards, S. M. Weir, C. G. Westlake, W. V. Whiteford.

F.R.C.S. Eng.—F. Langford, M.B., Ch.B. Bristol.

L.D.S., R.C.S.—*Final : Part I. only :* A. G. Batten, H. B. Smith, R. G. J. Tovey. *Parts I. and II. :* A. Jacobs.

L.S.A.—*Primary Examination. In Anatomy :* L. P. Gregory.

APPOINTMENTS.

Bristol Royal Infirmary. Assistant Surgeon : W. A. Jackman, F.R.C.S. Edin.

Bristol General Hospital. Ear, Nose and Throat Department. Assistant Surgeon : O. O. Popper, F.R.C.S. Edin.

Cossham Hospital. Ear, Nose and Throat Department, Surgeon : O. O. Popper, F.R.C.S. Edin.

